# NON-pharmacological Approach Less Invasive Surfactant Administration (NONA-LISA) trial: protocol for a randomised controlled trial

**DOI:** 10.1038/s41390-023-02998-0

**Published:** 2024-01-11

**Authors:** Niklas Breindahl, Tine Brink Henriksen, Christian Heiring, Emma Therese Bay, Jannie Haaber, Tenna Gladbo Salmonsen, Emma Louise Malchau Carlsen, Gitte Zachariassen, Peter Agergaard, Anne-Cathrine Finnemann Viuff, Lars Bender, Martin Grønnebæk Tolsgaard, Lise Aunsholt

**Affiliations:** 1 Department of Neonatal and Paediatric Intensive Care, Copenhagen University Hospital, Rigshospitalet, Copenhagen, Denmark; 2 Department of Clinical Medicine, University of Copenhagen, Copenhagen, Denmark; 3 Department of Paediatrics and Adolescent Medicine, Aarhus University Hospital, Aarhus, Denmark; 4 Perinatal Research Unit, Department of Clinical Medicine, Aarhus University, Aarhus, Denmark; 5 Hans Christian Andersen Children’s Hospital, Odense University Hospital and University of Southern Denmark, Odense, Denmark; 6 Division of Neonatology, Department of Paediatric and Adolescent Medicine, Aalborg University Hospital, Aalborg, Denmark; 7 Copenhagen Academy for Medical Education and Simulation (CAMES), Copenhagen University Hospital, Rigshospitalet, Copenhagen, Denmark; 8 Department of Obstetrics, Copenhagen University Hospital Rigshospitalet, Copenhagen, Denmark; 9 Department of Veterinary and Animal Science, University of Copenhagen, Copenhagen, Denmark

## Abstract

**Introduction:**

Using pre-procedure analgesia with the risk of apnoea may complicate the Less Invasive Surfactant Administration (LISA) procedure or reduce the effect of LISA.

**Methods:**

The NONA-LISA trial (ClinicalTrials.gov, NCT05609877) is a multicentre, blinded, randomised controlled trial aiming at including 324 infants born before 30 gestational weeks, meeting the criteria for surfactant treatment by LISA. Infants will be randomised to LISA after administration of fentanyl 0.5–1 mcg/kg intravenously (fentanyl group) or isotonic saline solution intravenously (saline group). All infants will receive standardised non-pharmacological comfort care before and during the LISA procedure. Additional analgesics will be provided at the clinician’s discretion. The primary outcome is the need for invasive ventilation, meaning mechanical or manual ventilation via an endotracheal tube, for at least 30 min (cumulated) within 24 h of the procedure. Secondary outcomes include the modified COMFORTneo score during the procedure, bronchopulmonary dysplasia at 36 weeks, and mortality at 36 weeks.

**Discussion:**

The NONA-LISA trial has the potential to provide evidence for a standardised approach to relief from discomfort in preterm infants during LISA and to reduce invasive ventilation. The results may affect future clinical practice.

**Impact:**

- Pre-procedure analgesia is associated with apnoea and may complicate procedures that rely on regular spontaneous breathing, such as Less Invasive Surfactant Administration (LISA).
- This randomised controlled trial addresses the effect of analgesic premedication in LISA by comparing fentanyl with a placebo (isotonic saline) in infants undergoing the LISA procedure. All infants will receive standardised non-pharmacological comfort.
- The NONA-LISA trial has the potential to provide evidence for a standardised approach to relief from discomfort or pain in preterm infants during LISA and to reduce invasive ventilation. The results may affect future clinical practice regarding analgesic treatment associated with the LISA procedure.

## Introduction

Respiratory distress syndrome (RDS) caused by surfactant deficiency remains a significant reason for neonatal mortality and short- and long-term morbidity in preterm infants.^1^ RDS usually develops during the first 24 h of the delivery, and most infants with RDS present with breathing difficulties and an increased need for oxygen supplementation within the first few hours of birth. Surfactant decreases alveolar surface tension and helps to keep the lungs aerated, allowing pulmonary gas exchange. Exogenous surfactant treatment of preterm infants with RDS reduces mortality and long-term morbidity.^2–4^

Less Invasive Surfactant Administration (LISA) is a strategy to administer surfactant to infants with worsening RDS despite optimised non-invasive ventilation. LISA aims to prevent alveolar collapse and avoid endotracheal intubation and invasive ventilation before, during, and after surfactant administration.^4–6^ LISA involves surfactant administration via a thin catheter placed in the trachea using laryngoscopy on a spontaneously breathing infant. Before LISA was introduced, surfactant was administered via an endotracheal tube followed by invasive ventilation and later via the INtubation-SURfactant-Extubation (INSURE) method with immediate extubation following surfactant administration. Studies have shown that using LISA reduces the incidence of intraventricular haemorrhage (IVH), mortality, the need for invasive ventilation, and the risk of bronchopulmonary dysplasia (BPD) in preterm infants.^7,8^ LISA is currently considered the preferred method of surfactant administration in spontaneously breathing infants according to the European Consensus Guidelines on Management of RDS,^4^ and LISA is increasingly used in NICUs worldwide.^9^

However, the need for analgesic premedication in LISA is debated. In a recent study of 153 LISA experts,^10^ 41% indicated no use of pre-procedure sedatives or analgesics, and 49% reported using fentanyl as a pre-procedure treatment. On the contrary, 4% indicated no use of non-pharmacological treatment. There is a delicate balance between the desired effect of analgesia reducing discomfort and pain associated with laryngoscopy as opposed to acute cardiovascular side effects and the risk of over-sedation and apnoea. This may potentially impede the procedure and result in the need for positive pressure ventilation, which may harm the surfactant-deficient lung.^11,12^ Observational data suggests the ease of performing LISA to be unaffected by whether opiates are used or not.^13^

The NONA-LISA trial (ClinicalTrials.gov, NCT05609877) compares LISA with saline to LISA with 0.5–1 mcg/kg fentanyl in infants born before 30 gestational weeks and evaluate the need for invasive ventilation via an endotracheal tube for at least 30 min (cumulated) within 24 h of the procedure. Secondary outcomes include the number of laryngoscopy attempts, duration of the procedure, modified COMFORTneo score during the procedure, bronchopulmonary dysplasia at 36 weeks, and mortality at 36 weeks.

## Methods

### Design and setting

**Fig. 1:**
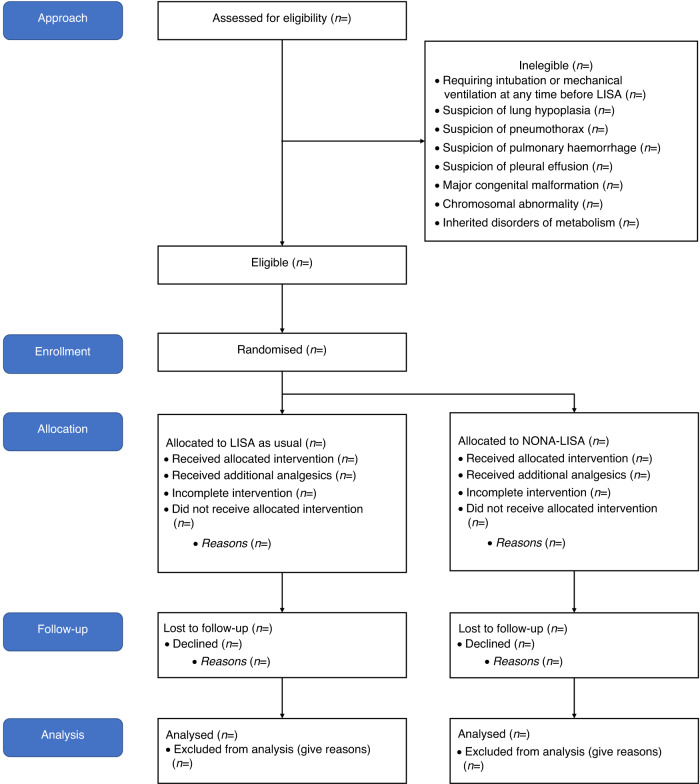
The Consolidated Standards of Reporting Trials (CONSORT) diagram of the NONA-LISA trial. Abbreviations: NONA-LISA NON-pharmacologic Approach Less Invasive Surfactant Administration, LISA Less Invasive Surfactant Administration, GA gestational age.

The NONA-LISA trial is a multicentre, blinded, randomised controlled trial of fentanyl 0.5–1 mcg/kg or placebo (isotonic saline), administered to infants born before 30 gestational weeks who meet the criteria for surfactant treatment by LISA. A total of 324 infants will be randomised in a 1:1 ratio to one of the two arms. They will be followed up at 36 weeks PMA and at hospital discharge (shown in Fig. 1). The trial protocol conforms with the Standard Protocol Items: Recommendations for Interventional Trials (SPIRIT),^14^ and the trial results will be reported in compliance with the Consolidated Standards of Reporting Trials (CONSORT) Statement.^15^ The trial will be initiated at (but not limited to) the Danish level III Neonatal Intensive Care Units (NICUs).

### Population

Infants will be eligible for inclusion if they are born before 30 gestational weeks at one of the trial sites and meet the criteria for first-choice surfactant treatment by LISA as described by Sweet et al.^4^: worsening babies with RDS and FiO_2_ > 0.30 on CPAP pressure ≥6 cm H_2_O. The primary respiratory support is CPAP in all four units.

Infants will be excluded if they meet any of the exclusion criteria; 1) suspicion of lung hypoplasia, 2) endotracheal intubation at any time before randomisation, 3) suspicion of pneumothorax, pulmonary haemorrhage or pleural effusion before LISA, 4) major congenital anatomical anomalies as described by the European Surveillance of Congenital Anomalies (EUROCAT).^16^

### Randomisation

Participants will be randomly assigned to the saline or fentanyl group in a 1:1 ratio using computer-generated random allocation sequences with permuted blocks of varying sizes (two and four). The randomisation sequence will be stratified by trial site and gestational age at birth (more or less than 28 completed gestational weeks). Once randomised in the Research Electronic Data Capture (REDCap) system, all entry data will automatically be transferred to the electronic case record forms (eCRF) linked to the infant’s unique identification number. The allocation sequence is pre-coded and generated from the randomisation programme. A person not involved in the trial will generate the allocation sequence. The clinical staff will enrol participants.

### Blinding

Blinding will be secured by the following means: The patient will be randomised after entering stratification variables in REDCAP. The randomisation sequence will be generated by one person unrelated to the study. The trial medication (fentanyl or isotonic saline) will be prepared once per day in sealed syringes with similar appearance and weight by the pharmacy or staff not involved in patient care. Thus, neither staff involved in screening or treatment of patients nor the parents will know the treatment allocation. Primary analyses will be performed blinded to the group allocation (Group A compared with Group B) and presented to all authors, who will agree on two alternative written interpretations before the randomisation code is unblinded to reduce the risk of interpretation bias.

### Interventions

Participants are randomised to receive LISA with fentanyl 0.5-1 mcg/kg (fentanyl group) or LISA with isotonic saline (saline group). All infants will receive standardised non-pharmacological comfort care before and during the LISA procedure (Online Supplement, Appendix A). All LISA procedures will be performed using video laryngoscopes according to the Hobart method.^2,3^ Both groups will receive the neonatal unit’s standard pre-procedure care (e.g., atropine and caffeine), which will not be standardised across trial sites. Naloxone may be administered at the clinician’s discretion and will not be used routinely. In both groups, the infant’s level of discomfort or pain will be monitored continuously during the procedure by vital signs and a modified COMFORTneo score (Online Supplement, Appendix A).^17^ Open-label analgesics can be administered to all participants at the clinician’s discretion by indications of non-tolerance (e.g., modified COMFORTneo score >14).

All administered medications will be registered. A single dose of 200 mg/kg porcine surfactant (Curosurf®, Chiesi Pharma AB, Italy) will be used in this trial.

### Outcomes

#### Primary outcomes

The primary outcome of this trial is the need for invasive ventilation, meaning mechanical or manual ventilation via an endotracheal tube for at least 30 min (cumulated) within 24 h of the procedure. Non-invasive ventilation (NIV) is not included in the primary outcome.

#### Secondary outcomes

The secondary outcomes include:

- Unique and composite outcome of death or moderate/severe BPD^18^ at 36 weeks PMA.
- Adverse events during the procedure (from the introduction of the laryngoscope blade into the oral cavity to the removal of the catheter) in terms of apnoea that require bag and mask ventilation, desaturation with an absolute decrease in peripheral oxygen saturation >20% from pre-procedure baseline, and bradycardia <100 BPM individual outcomes.^19^
- Pain or discomfort during the procedure (according to modified COMFORTneo score >14^17,20,21^).
- Highest modified COMFORTneo score during the procedure.
- Need for a second dose of surfactant.
- Incidence of LISA procedures resulting in the INSURE procedure.
- Incidence of observed surfactant reflux.
- Incidence of observed injury to the upper airway.
- Incidence of observed injury to the lower airway.
- Incidence of invasive ventilation within 48 h after LISA.
- Cumulated duration of invasive ventilation during hospitalisation.
- Cumulated duration of any non-invasive respiratory support during hospitalisation.
- Cumulated duration of any respiratory support during hospitalisation.
- Procedural duration from the introduction of the laryngoscope blade over the lips to the removal of the catheter.
- Number of attempts to visualise vocal cords (laryngoscopy) where the laryngoscope is completely removed from the oral cavity between attempts.
- Incidence of pneumothorax within 48 h after LISA.
- Incidence of massive pulmonary haemorrhage within 48 h after LISA (aspiration of haemorrhagic secretions from the trachea concurrent with the need for escalated respiratory support).^22^
- Duration of hospitalisation.
- Morbidities in terms of necrotising enterocolitis (according to the radiographic signs of Bell’s Staging Criteria),^23^ treatment-demanding retinopathy of prematurity, intraventricular haemorrhage grade 3–4 and periventricular leukomalacia (according to the Papile classification)^24^ as individual outcomes.

#### Data collection

Baseline characteristics will include infant gestational age at birth, delivery mode, singleton or multiple births, birth weight, sex, APGAR score (1, 5, and 10 min after delivery), resuscitation measures during the first 30 min after birth, first blood gas, umbilical arterial and venous pH, standard base excess, and lactate. Information on vital signs (oxygen saturation, FiO_2_, and heart rate) will be collected 15 min before and after the procedure. Information on treatment with antibiotics, fluids, vasopressors, and early caffeine before the LISA procedure will be recorded. We will calculate the time from meeting the criteria for surfactant treatment until the procedure starts. We will also include maternal information regarding smoking status, diseases (diabetes mellitus, amnionitis as per the obstetrician, preeclampsia, and eclampsia), and antenatal administration of steroids, magnesium sulphate, indomethacin, and antibiotics, including timing.

A generic procedure description will be created for the clinician to use in the medical records to ensure that all information, including modified COMFORTneo score before the procedure, administration of atropine, early caffeine and naloxone, and indications for additional fentanyl administration and cumulated dosage, is recorded uniformly.

If the infant is discharged to a step-down unit, information will be retrieved from that unit.

#### Data management

Study data will be collected and managed using REDCap electronic data capture tools hosted at the Capital Region of Denmark.^25^ A password will protect access to the online forms and dataset, and infants will be identified by number only to protect confidentiality before, during, and after the trial. A clinical trial coordinator will be responsible for monitoring the study’s progress and the data’s completeness.

#### Patient and public involvement

A parent representative provided feedback resulting in minor revisions of the parent information material and consent forms before trial initiation (Online Supplement, Appendices B and C). A parent representative will also be involved in the dissemination plans of the trial. However, parents were not involved in the study design and will not be involved in recruiting or data collection during the study.

#### Sample size estimation and inclusion timeline

The sample size was calculated according to the primary outcome. Previous studies on preterm infants born before 30 gestational weeks report a frequency of invasive ventilation after the LISA procedure from 33%^26^ and 41%^27^ up to 75%.^28^ Based on the incidence of invasive ventilation following LISA, we anticipate that the primary outcome will have an incidence of around 45% in the fentanyl group in our trial. Considering an alpha of 0.05 and a power of 0.8 to detect an anticipated incidence of about 30% for the saline group, this trial will enrol 324 infants in a 1:1 ratio. Based on an article by Wiingreen et al., ~123 infants are born before 30 gestational weeks each year in Denmark with the need for surfactant.^29^ The inclusion will last three to four years, considering an expected inclusion rate of 80%.

#### Statistical methods

Baseline characteristics will be presented for each group in the trial population. Categorical variables will be presented as frequencies (counts and percentages). Continuous variables will be presented as medians with interquartile ranges [IQR] or as means with standard deviations [SD].

The primary analysis will use the intention-to-treat principle. A secondary analysis will use the per-protocol principle and exclude infants if they receive additional analgesics associated with the procedure.

The primary outcome will be reported as frequencies (counts and percentages) per treatment group with unadjusted absolute risk difference and relative risks with 95% confidence intervals (CI). The number needed to treat for benefit or harm will be reported if the results of the primary analyses show a statistically significant difference between the two exposure groups.

Secondly, a logistic regression model will be performed adjusting for inclusion site and gestational age (<28 weeks vs more than 28 weeks) and the following independent covariates, which are predictors of assisted ventilation and length of ventilation:^30^ repeat doses of prenatal corticosteroids, 5-min APGAR scores, sex, admission illness severity (according to the Score for Neonatal Acute Physiology/SNAP score^31^), oxygenation index, and small-for-gestational-age status.^32^

All secondary dichotomous outcomes will be described and analysed per the same strategy as the primary outcome. At the same time, mean and percentage differences will be calculated for continuous outcomes.

All analyses will be conducted by use of the statistical software R Studio. Two-sided P values less than 0.05 will be considered statistically significant.

## Discussion

This randomised controlled trial compares infants exposed to the LISA procedure with pre-procedure fentanyl or placebo (isotonic saline) by the risk of invasive ventilation, meaning mechanical or manual ventilation via an endotracheal tube, for at least 30 min (cumulated) within 24 h of the procedure. All infants will receive standardised non-pharmacological comfort care before and during the LISA procedure (Online Supplement, Appendix A). In this trial, we plan to test the hypothesis that there is no difference between the need for invasive ventilation within 24 h of the LISA procedure performed with or without pre-procedure analgesia (i.e., fentanyl).

The LISA procedure is performed worldwide without consensus regarding non-pharmacological and pharmacological approaches. There is clinical equipoise regarding the positive and negative effects of pharmacological analgesic treatment when performing the LISA procedure. There is a delicate balance between the desired effect of reduction in pain and discomfort and the risk of over-sedation and apnoea requiring positive pressure ventilation.^11,12^ Comfort and pain relief using a non-pharmacological approach has yet to be thoroughly investigated in the context of LISA and how it compares with pharmacological analgesic treatment. Some studies indicate that a non-pharmacological approach, including facilitated tucking,^33^ swaddling,^34^ and skin-to-skin care^35^ compared to pharmacological analgesic treatment, may reduce the incidence of apnoea and, ultimately, the use of invasive ventilation,^20,21^ while achieving the same level of comfort. Hence, some centres make the first LISA attempt without any premedication, as studies report that the LISA procedure is generally well tolerated without analgesia,^1,36,37^ especially in preterm infants.^38^ Therefore, some studies advise using pharmacological agents only if non-pharmacological methods are insufficient to ensure patient comfort.^39^ It is essential to investigate pharmacological and non-pharmacological approaches to reduce pain, discomfort, and adverse effects due to medication during neonatal procedures, including the LISA procedure, as the insertion of a laryngoscope and intratracheal administration of surfactant obviously contradicts the term “less invasive”.^40^

As a safety precaution in the NONA-LISA trial, the staff will continuously monitor the infant’s level of discomfort or pain during the procedure by an objective score (modified COMFORTneo). The COMFORTneo score is the national standard for assessing pain and discomfort in newborns. Most NICU nurses are COMFORTneo certified. Previous studies have demonstrated the ability of the COMFORTneo score to assess pain and discomfort during the LISA procedure.^20,21^ However, the COMFORTneo score formally requires a two-minute assessment of the infant and was not designed for intra-procedure assessments as planned in the NONA-LISA trial. To avoid interrupting the procedure flow, performing the score over 2 min is not feasible. Thus, intra-procedure assessments of COMFORTneo in this trial will be based on the standard COMFORTneo items assessed faster than 2 min. The use of COMFORTneo in this trial is described in detail in Online Supplement, Appendix A. Importantly, if the modified COMFORTneo score is ≥14 during the procedure, the procedure may be paused, and non-pharmacological measures must be improved. If improved non-pharmacological measures are ineffective, the team may opt for open-label fentanyl administration. The infant will still be given the highest level of care and attention by the staff.

This trial has certain limitations. The pragmatic design of this trial allowing fentanyl dosages between 0.5–1.0 mcg/kg will imply difficulty interpreting the specific results, as this trial will not have the necessary power to determine causality between an exact dosage of fentanyl and adverse events (e.g., apnoea). Despite this, a pragmatic design was necessary to ensure participation from all feasible Danish trial sites. As the dosing interval reflects clinical practice, it ensures generalisability and relevance for neonatal intensive care outside trial settings.

Non-pharmacological measures in neonatal care are complex and multifactorial, where all factors are relevant for the potential success of the intervention. Several studies have shown synergy when combining several non-pharmacological measures. However, there is no evidence favouring one specific approach. This study will use a protocolised non-pharmacological approach to standardise the treatment across inclusion sites.

The incidence of invasive ventilation following LISA varies significantly in other studies, and the anticipated incidences in the fentanyl and saline groups may cause an inadequate sample size to achieve the necessary power. However, compared to ongoing clinical trials like PRELISA (NCT05065424), we expect to include more than five times as many infants in the NONA-LISA trial. Other trials like the PROLISA (NCT04016246) include more infants but compare propofol to placebo.

The NONA-LISA trial received approval for deferred consent, which may significantly improve the inclusion rates. Nevertheless, with all the Danish NICUS as inclusion sites, the inclusion period will last up to four years, making the NONA-LISA trial vulnerable to changes in best practices regarding surfactant treatment. We expect to include other trial sites.

## Conclusion

In conclusion, the NONA-LISA trial has the potential to provide evidence for a standardised approach to relief from discomfort or pain in preterm infants during LISA (NONA-LISA) and to reduce the need for invasive ventilation during the first day after the procedure.

## Supplementary information

Appendix

## Data Availability

The datasets generated during and analysed during this trial are available from the corresponding author upon reasonable request.
