# Supplementary material for: NON-pharmacological Approach Less Invasive Surfactant Administration (NONA-LISA) trial: protocol for a randomised controlled trial
Source: Pediatr Res. 2024 Jan 11;96(4):1084–9. doi: 10.1038/s41390-023-02998-0 (PMC11502479; doi:10.1038/s41390-023-02998-0)
Supplement: Supplementary file 1 — Appendix [file 41390_2023_2998_MOESM1_ESM.pdf]

Appendix A: Protocolised non-pharmacological approach for both treatment arms in the NONA-LISA trial

Distribution of roles

In both groups, the LISA procedure must be undertaken by 3-4 staff members: 1) The neonatologist guiding the video laryngoscope, 2) Neonatal nurse 1 (certified in COMFORTneo scoring) taking care of non-pharmacological measures and assessment of the modified COMFORTneo score, 3) Neonatal nurse 2 administering the medication and assisting during the procedure, 4) One person (e.g., a doctor, nurse, or other hospital staff) taking care of the procedure registration including the COMFORTneo score assessed by neonatal nurse 1.

The main features of the procedure

1. The infant's comfort and the possibility to complete the LISA procedure with little or no discomfort are of highest priority.
2. A COMFORTneo score is made immediately before the procedure. To avoid interrupting the procedure flow, performing the score over 2 minutes will be impossible. Thus, intra-procedure assessment of COMFORTneo involves all items but a scoring faster than the usual two 2 minutes. The procedure can start 3-5 minutes after administration of trial medication and only with a modified COMFORTneo score between 8-13 and sufficient respiratory drive. The specific assessment is made by neonatal nurse 1 and the neonatologist in collaboration.
3. The infant's level of pain and discomfort are assessed during the procedure (from the introduction of the laryngoscope blade into the oral cavity to the removal of the catheter). The highest COMFORTneo score will be registered. If the score is ≥14 during the procedure, the procedure may be paused, and non-pharmacological measures must be improved. If improved non-pharmacological measures are insufficient, open-label fentanyl must be administered. Once surfactant is administered, the rate of administration can be slowed down, or the administration can be paused, while the catheter remains in the trachea.
4. Start the recording on the video laryngoscope. Start by filming the child's personal identification number and let the video run throughout the duration of the laryngoscopy. Stop recording when the laryngoscopy is finished, i.e., when the catheter is in place.

Non-pharmacological measures

- Create a quiet and calm atmosphere (e.g., close the door, adjust the light to avoid bright light in the infant's face, and use quiet voices).
- Ensure an appropriate temperature.
- The infant is positioned horizontally in the incubator in a lateral or prone position and contained using a 'nest'.
- The infant is swaddled. Arms and legs are supported in a flexed position close to the body.
- The infant is monitored with oxygen saturation and ECG.
- The nurse assesses whether the infant is offered mother's milk or oral sucrose. Administration depends on the infant's ability to cooperate. Mother's milk or 0.05-0.1 ml of oral sucrose (whichever is preferred) is administered 2 min before the procedure.
- Immediately before the procedure, the infant is gently moved into a supine position, and the optimal support is placed under the shoulders.
- Neonatal nurse 1 supports the infant with arms and legs flexed close to the body and assesses the modified COMFORTneo score. See number 2 above.
- Next, the trial medication is administered, and the procedure begins.
- Neonatal nurse 1 monitors the infant's level of pain and discomfort during the procedure. The highest COMFORTneo score will be registered. See number 3 above.
- After the procedure, the infant is placed in the most optimal position to support spontaneous breathing, and neonatal nurse 1 can let go of the child when it is comfortable.

Appendix B: Parental information (English)

Title of the trial:

NONA-LISA: NON-pharmacological Approach Less Invasive Surfactant Administration

An investigator-initiated, multicentre, pragmatic, parallel-group, blinded RCT conducted at four university hospitals across Denmark evaluating the effect of surfactant administration in premature infants using the LISA procedure with or without fentanyl.

Dear parent

You receive this leaflet because your child was born very early and needs intensive care. We understand that this may be a stressful time for you and that taking in information at this time may be difficult. This leaflet gives you information about a clinical trial called NONA-LISA. We are conducting this trial to investigate if surfactant administration using the LISA procedure without fentanyl (a short-acting, highly potent opioid) can optimise the effect and improve the outcome of infants in the same situation without inducing pain or discomfort.

Because the trial must begin as soon as your child meets the criteria for surfactant treatment, we have already randomised your child.

We now ask for your permission to continue having your child in the trial. You are free to decide.

What does trial participation involve?

If a child gets randomised, i.e., is randomly selected, to the control group, he or she has already received the usual LISA procedure, including fentanyl and other standard pharmacological treatment and support given in the neonatal intensive care unit. During the procedure, the attending neonatologist (doctor) and the neonatal nurse have continually monitored your child's response to the procedure and provided more fentanyl if indicated.

If, on the other hand, a child is randomised to the intervention group, he or she has already received the LISA procedure, including all usual pharmacological treatment and support except fentanyl. During the procedure, the attending neonatologist (doctor) and the neonatal nurse have continually monitored your child's response to the procedure and provided fentanyl if indicated.

All future treatment and care in the neonatal intensive care unit will be performed according to usual practice and will not differ between the control and the intervention group. No extra examinations are done, but data from your child's medical records will be used to calculate the trial result. The trial includes infants from neonatal intensive care units all over the country. The Committee on Health Research Ethics in the Southern Region of Denmark approved the trial.

What are we trying to find out?

Approximately 50% of physicians perform the Less Invasive Surfactant Administration (LISA) procedure using fentanyl, a powerful synthetic opioid similar to morphine but 50 to 100 times more potent. Unfortunately, fentanyl can cause apnoea and procedural failure.
We want to know if the LISA procedure using a non-pharmacologic approach without fentanyl can reduce the need for mechanical ventilation during the first 24 hours after the procedure.

The above contains the essential information. On the following pages, you can read more details and the standard conditions for participating in research projects. Do not hesitate to ask questions.

What information do we need from the child's medical records?

All information will be readily available in the medical records without needing further examinations (e.g., blood samples, clinical examinations etc.).
We will collect information from the mother's and child's medical records regarding the progress and treatment during pregnancy, labour, and stay at the neonatal intensive care unit. This information will be used to compare the control and intervention group.

How can we monitor the child's level of discomfort during the procedure?

In both the control and the intervention group, the infant's level of discomfort or pain during the procedure will be monitored continuously by vital signs (e.g., pulse and blood pressure) and behaviour. Both groups will receive treatment from experienced teams of neonatal nurses and neonatologists (doctors), and additional analgesics (fentanyl) will be provided at the clinician's discretion. Staff will give your child the highest level of care and attention.

How will this change the treatment of my child?

Both procedures will follow the national guideline related to LISA using video laryngoscopes. Infants will receive standard care in the neonatal intensive care unit. All future treatment and care in the neonatal intensive care unit will be done according to usual practice and will not differ between the control and the intervention group. There are no extra examinations done.

How will the research be done?

NONA-LISA is a randomised clinical trial. Many treatments are tested by randomised clinical trials, which is the most reliable way to determine if they are effective.

A randomised clinical trial means that the decision about whether the child will receive LISA with or without fentanyl will be determined by chance, like the toss of a coin. This means that half of the infants participating in the NONA-LISA trial will receive LISA without fentanyl (intervention group). In contrast, the other half will receive LISA as usual, i.e., with fentanyl (control group). The neonatologist and the neonatal nurse will initially be blinded to treatment with analgesia or a placebo (isotonic saline solution). However, additional doses of analgesia (fentanyl) will be given at the clinician's discretion and will not be blinded. To evaluate the intervention effect, we will include 324 babies from the four highly specialised neonatal intensive care units in Copenhagen, Odense, Aarhus, and Aalborg. All necessary information will be collected in the days/weeks after the procedure through your child's medical record until 36+0 weeks of gestational age.

Are there risks?

There is clinical equipoise regarding the effect of pharmacological analgesic treatment when performing the LISA procedure, as there is a delicate balance between the desired effect of analgesia reducing discomfort and, on the other hand, the risk of over-sedation and apnoea. While analgesia may increase the chance of a successful procedure, it may also compromise the infant's respiratory drive, which is a prerequisite for LISA. Using video laryngoscopes has reduced the undesired effects of laryngoscopy (pain or discomfort). Now, video laryngoscopes are used in all neonatal intensive care units in Denmark. Other studies report that the LISA procedure is generally well tolerated without analgesia (fentanyl), and some centres will make the first LISA attempt without any premedication. The comfort and pain relief using a non-pharmacological approach

compared with pharmacological analgesic treatment has yet to be thoroughly investigated. There is a significant knowledge gap regarding one being superior or equal to the other. Some studies indicate that a non-pharmacological approach, including facilitated tucking, swaddling, and skin-to-skin care compared to pharmacological analgesic treatment, may reduce the incidence of apnoea and, ultimately, the use of positive pressure ventilation, including mechanical ventilation.

The research could provide valuable knowledge and potentially lead to a higher success rate of surfactant administration in those needing surfactant treatment.

However, we have yet to find out at present. That is why it is necessary to test it properly in a randomised trial to clarify risks and benefits before taking the NONA-LISA procedure into routine use. So, as in all research, there may be unforeseen risks. All severe adverse reactions will be reported as a part of the research to ensure that we learn as much as possible from them.

All observations, treatments and additional monitoring will be as usual in the neonatal department.

The investigators will access your child’s hospital records for trial purposes, i.e., trial completion, surveillance, and control of trial in the weeks after the procedure until 36+0 weeks of gestational age.

Any information we collect from the medical records will be kept confidential and secure according to the Danish Data Protection Act following approval by the Danish Data Protection Agency. Only authorised people involved in the trial will have access to data from your child.

Will there be follow-up studies?

We plan to roll out an additional study to follow up on your child's progress. Therefore, you may be contacted within the first two years of your child's life. Follow-up may include a doctor's examination, a questionnaire, or a developmental test. You will be asked for separate consent for this or any other study that may be relevant for infants participating in the NONA-LISA trial.

Does my child have to continue in NONA-LISA?

No. It is your decision whether your child should continue in the NONA-LISA trial. If you decide to let your child continue in the trial, you may change your mind later without giving a reason and with no consequences for your child's future care.

If you decide that you do not want your child to continue in the NONA-LISA trial, he or she will still be given the highest level of care and attention by staff.

When is the trial terminated?

An external committee monitors the trial. If there are unexpected critical problems or the trial cannot answer the questions it was designed to answer, the trial may be stopped.

Will I be told the results of NONA-LISA if we join?

Yes. If you decide that you would like your child to join NONA-LISA, we will keep in touch with you to tell you the trial results when they become available if you wish.

The results from the NONA-LISA trial will, regardless of whether positive, negative, or inconclusive, be published in a publicly accessible journal.

What will happen to the data you have collected regarding my child?

The persons involved in this trial have no economic interest in any aspect of the trial. This trial is solely made to improve the care of infants born too early. Therefore, to optimise the value of your child's participation, we will store your child's anonymised data for at least ten years so that other researchers may use them to examine other research questions in the same field.

How is the trial funded?

The trial was planned by the NONA-LISA trial group, consisting of academic neonatologists, and is partially supported by Chiesi Pharma AB. Chiesi Pharma AB did not influence the trial design, data collection, analysis, interpretation, writing or decision to submit the manuscript for publication. Other sponsors may follow. All financial support will be deposited in a bank account belonging to the department initiating the study.

The investigators have no personal attachment to Chiesi Pharma AB or any other organisation/company interested in surfactant treatment.

Families will not receive payment for participation.

Thank you!

Thank you for taking the time to read this leaflet. Please ask your doctor or neonatal nurse if you would like more information about the NONA-LISA trial. Attached to this leaflet you will find two additional leaflets entitled: 1) “Forsøgspersonens rettigheder I sundhedsvidenskabelige forskningsprojekter” and 2) ”Før du beslutter dig”.

Contact information for trial sponsor

Lise Aunsholt, MD, PhD, Ass. Professor
Address: *Department of Neonatal and Pediatric Intensive Care, Copenhagen University Hospital, Rigshospitalet*, Blegdamsvej 9, 2100 Copenhagen, Denmark
Telephone:
Mail:

Appendix C: Informed consent form (English)

Title of the trial: The NON-pharmacological Approach Less Invasive Surfactant Administration trial

Declaration from the parents:

I/we have been fully informed by the attending doctor OR neonatal nurse (investigator) of the trial's potential favourable and adverse effects.

I/we understand the aims, randomisation procedure, intervention, significance, and relevance of the NONA-LISA trial.

I/we have been informed that participation is voluntary and that I/we can withdraw my/our consent at any time without providing reasons and without incurring any disadvantages for my/our child or myself/us.

I/we have received a copy of this declaration of consent (signed) and the accompanying parental information and read and understood the text.

I/we confirm with my/ours signature(s) that I am/we are willing to let my/our child participate in the NONA-LISA trial.

Participant/child’s name:

____

Name parent 1:

____

Signature: Date:

____

Name parent 2:

____

Signature: Date:

____

Do you wish to stay informed about the trial results?

- □ Yes
- □ No

Trial site investigator (or delegated personnel) providing the parental information:

I hereby confirm that the participant's parent/parents have been given verbal and written information about the trial. I am convinced the parent/parents have been given sufficient information and that the information is understood, such as informed consent can be given.

Name:

Signature: Date:

ID-number (Committee on Health Research Ethics): H-21078489

Trial sponsor:

Lise Aunsholt, MD, PhD, Ass. Professor
Address: *Department of Neonatal and Pediatric Intensive Care, Copenhagen University Hospital, Rigshospitalet*, Blegdamsvej 9, 2100 Copenhagen, Denmark
Telephone:
Mail:

Appendix D: Justification of inclusion via “deferred consent”

In most situations, preterm delivery is an unforeseen medical emergency, and the preterm infant is often in need of intensive care treatment from the very beginning, leaving the parents in a very distressing situation. Further complicating this is that interventions often need to be initiated within a short time frame to have the desired effect in neonatal emergencies. Obtaining prior informed consent before providing a specific treatment may delay the treatment to an extent where the neonate could miss out on a treatment that could have been of benefit.

It is well-described how the distress and time constraints associated with obtaining consent for emergency neonatal research may compromise understanding and voluntariness, essential components of adequately informed consent[56–60]. Despite these obstacles, clinical research in emergencies is paramount. This is the only way to obtain new data that can guide evidence-based practice, potentially saving lives or reducing the risk of certain conditions or procedures.

The *waiver of consent* or *deferred consent* is a well-recognised approach to the difficulties encountered in the enrolment of patients in studies of emergency procedures. Deferred consent was first described in 1980 and has now been used in many trials that have yielded important information that has changed clinical practice [61]. Deferred consent involves enrolling patients into clinical trials without seeking prior consent. Consent is obtained later, in paediatrics, usually from the parents or a legal guardian; in this case, the patient can continue participating in the trial. Otherwise, the patient and data are withdrawn from the trial. In Copenhagen, we have recently conducted a trial on infants using this approach (VEK protocol number: H-3-2013-182) [62].

In 2008 deferred consent was allowed in the UK when the following criteria were met: treatment is required urgently, urgent action is required for the trial, and it is not reasonably practical to obtain consent. The UK Medical Research Council state that "*provided that the specific approval of a research ethics committee has been obtained for the project overall, it is ethical to carry out research involving children on occasions of extreme urgency without obtaining prior consent*” and that “*the parents and child must be informed about the research as soon as possible afterwards and their consent for future involvement sought*”[63]. They also state that the research should only be carried out to improve understanding of the subject's condition, that it entails only minimal risk and that it must be made clear to the parent/child that they can withdraw from the study at any point.

This is also supported by Article 19 of the 'Additional protocol on the Convention of Human Rights and Biomedicine on Biomedical Research' affirmed by the Council of Europe in 2004[64]. The

same principles apply in the Danish legislation (Komitélovens §11 and §12) concerning clinical studies involving patients in emergencies where informed consent cannot be obtained before enrolment[65]. Studies have shown that parents and caregivers (in particular those with prior experience) generally have a positive attitude towards deferred consent[59, 66, 67].

We believe that the NONA-LISA trial satisfies these criteria for the following reasons:

- Both parents may be unavailable due to the urgency of extreme preterm birth.
- Parents may be unprepared in acute delivery cases without prior knowledge of the risks of preterm birth and treatment options.
- Parents may be unable to provide informed consent due to emotional distress or if undergoing anaesthesia for the delivery or complications related to the delivery. The mother herself is a patient, and the father may be facing several threats to his family.
- Deferred informed consent, although incompletely providing the opportunity for parents to opt-out, allows proper time for information, reflection, and decision.
- Seeking valid prior informed consent is time-consuming and could delay time-critical interventions, thereby reducing the effect of the intervention.
- Vulnerable populations, such as critically ill children, should not be denied the opportunity to participate in research due to difficulties in the informed consent process. This is an essential issue in pediatric emergency care since multiple treatments routinely administered as part of clinical guidelines are lacking solid evidence.
- There is a clear objective of the research, which could reduce the burden of morbidity and risk of death to the group of patients represented by the study population.
- There is clinical equipoise regarding the effect of pharmacological analgesic treatment when performing LISA. This research could provide valuable knowledge and potentially lead to a higher success rate of surfactant administration in those needing surfactant treatment.
- A low participation rate is a problem for the feasibility of the trial and the quality and generalizability of its results.
- This research may lead to a change in practice and decrease LISA failure compared to routine treatment, which is clinically meaningful.
- We will inform parents as soon as is practicable that their infant was enrolled in the study and ask their permission to collect their infant’s information giving them ample time to consider participation.

- We will inform parents that they may withdraw their child from the study at any time without explanation.

Details regarding obtaining consent after inclusion

If the Committee on Health Research Ethics approves inclusion via deferred consent, the following procedure will be followed:

- Written informed consent will be sought as soon as possible after enrolment.
- The parents will receive the parent information sheet and consent form (available from the principal investigator upon request) from an investigator (or investigator's delegate, such as a qualified physician or neonatal nurse connected to the trial). The investigator will verbally explain the study and the written material in plain language.
- Every effort will be made to conduct the conversation with the parents in a calm atmosphere away from the clinical area without interruptions. Accordingly, the investigator informing the parents will ensure that phone calls etc. will be forwarded to colleagues.
- Because the intervention has already been done at the time of information and outcome calculations are made from routine information obtained from the medical chart, there will be time to meet with the parents several times to clarify any questions.
- At the time of the first contact, the parents will be informed verbally and in writing via the information material, that they have the right to invite a family member, friend, or another person of their choice to participate and support them at the information meeting.
- The parents will be given options to ask any question they may have. They will also be explained their legal rights. In addition to the information sheet about the study, a copy of "Forsøgspersoners rettigheder i et sundhedsvidenskabeligt forskningsprojekt" will be handed to them[68].
- Participation is voluntary, and parents have the right to refuse or withdraw consent to participate in the study without warning or explanation. This decision will not influence the treatment of their child.

Appendix E: Charter for Data Monitoring and Ethics Committee (DMEC) for the NONA-LISA trial

**1. INTRODUCTION**	
Name of the trial	**NONA-LISA trial**: The NON-pharmacological Approach Less Invasive Surfactant Administration
Registration numbers (clinicaltrials.gov)	**NONA-LISA trial**: NCT05609877
Objectives of trial, including interventions being investigated:	**NONA-LISA trial**: This multicentre, blinded, randomised controlled trial will include 324 infants born before 29 completed gestational weeks meeting the criteria for surfactant treatment by LISA. Infants will be randomised to receive surfactant by the LISA procedure using identical volumes of an isotonic saline solution or 1 mcg/kg fentanyl. Both groups will be handled using an identical structured non-pharmacological approach. Additional analgesics will be provided at the discretion of the clinician guided by structured monitoring and assessment of the child. The primary outcome is the need for endotracheal intubation and mechanical ventilation for at least 30 minutes (cumulated) within 24 hours after the procedure. Secondary outcomes are numerous and include a structured, validated pain and discomfort score during the procedure, mortality at 36 weeks, and bronchopulmonary dysplasia at 36 weeks.
Website:	**FAST2 trial trial**: N/A
Outline of the scope of the Charter:	The purpose of this document is to describe the roles and responsibilities of the Data Monitoring and Ethics Committee (DMEC) for the NONA-LISA trial, including the timing of meetings, methods of providing information to and from the DMEC to the trial study groups, frequency and format of meetings, safety and statistical issues, and relationships with other committees.
Coordinating investigator:	**NONA-LISA trial**: Niklas Breindahl, MD PhD student

Members of the study groups:	**NONA-LISA study group**<br>Niklas Breindahl, MD, Rigshospitalet, Denmark (coordinating investigator)<br>Tine Brink Henriksen, MD, Professor in Pediatrics, Aarhus University Hospital, Denmark (site investigator and co-supervisor)<br>Christian Heiring, MD, Consultant in Pediatrics, Rigshospitalet, Denmark (site investigator)<br>Emma Therese Bay, MSc, Rigshospitalet, Denmark (site investigator)<br>Emma Louise Malchau Carlsen, MD, Associate Professor in Pediatrics, Rigshospitalet, Denmark (site investigator)<br>Gitte Zachariassen, MD, Professor in Pediatrics, Odense University Hospital, Denmark (site investigator)<br>Peter Agergaard, MD, Consultant in Pediatrics, Aarhus University Hospital, Denmark (site investigator)<br>Anne-Cathrine F. Viuff, MD, PhD, Senior Staff Specialist in Pediatrics, Aalborg University Hospital, Denmark (site investigator)<br>Lars Bender, MD, Senior Staff Specialist in Pediatrics, Aalborg University Hospital, Denmark (site investigator)<br>Martin Tolsgaard, MD, Professor in Medical Education, CAMES, Rigshospitalet, Denmark (co-supervisor)<br>Lise Aunsholt, MD, PhD, Consultant in Pediatrics, Rigshospitalet, Denmark (sponsor and supervisor)
**2. ROLES AND RESPONSIBILITIES**	
A broad statement of the aims of the committee:	The overall responsibility of DMEC is to safeguard the interests of the trial participants, assess the safety issues that may arise during the trial, and monitor the overall conduct of the trial.
Terms of reference:	The DMEC will receive and review the progress and accruing data of the NONA-LISA trial and provide advice on the conduct of the trial to the trial study group.<br>The DMEC will inform the trial study group if, in their view:

	The information they receive shows (a) evidence that the intervention demonstrates substantial harm to the patient and (b) evidence that might be expected to influence many clinicians' patient management.
Specific roles of DMEC:	Review of the trial's progress, including updated figures and numbers on overall recruitment, compliance, data quality, and safety data.<br>A selection of specific aspects could be compiled from the following list blinded to treatment:<br>• Assess data quality, including completeness (and by so doing, encourage the collection of high-quality data).<br>• Monitor recruitment figures and losses to follow-up.<br>• Monitor adherence with the protocol by participants and investigators.<br>• Monitor trial conduct – organisation and implementation of the trial protocol (the DMEC should only perform this role in the absence of other trial oversight committees).<br>• Monitor evidence for treatment harm (e.g., AEs and SAEs). All SAEs must be reported within 24 hours to the trial sponsor, who will report the SAE to the National Ethics Committee and the DMEC within 24 hours. The other coordinating centres will be informed within 3 working days. All AEs must be reported to the trial sponsor every month, who will report the AE to the DMEC.<br>• Decide whether to recommend that the trial continues to recruit participants or whether recruitment should be terminated either for everyone or for some participant subgroups.<br>• Suggest additional data analyses.

	• Advise on protocol modifications suggested by investigators or sponsors (e.g., to inclusion criteria, trial endpoints, or sample size)<br>• Monitor planned sample size assumptions.<br>• Monitor compliance with previous DMEC recommendations.<br>• Consider the ethical implications of any recommendations made by the DMEC.<br>• Assess the impact and relevance of external evidence.
**3. SPECIFICATIONS**	
Whether the DMEC will have input into the protocol:	All potential DMEC members will review the protocol before agreeing to join the committee. Before recruitment begins, the NONA-LISA trial will have undergone review by the sponsors, Christian Heiring and Lise Aunsholt, respectively (Department of Neonatology, Copenhagen University Hospital Rigshospitalet), scrutiny by other trial committees and a research ethics committee. By accepting the invitation to join the DMEC, it is essential that the members have no significant reservations about the trial (e.g., the protocol or the logistics).<br>DMEC members should be independent and constructively critical of the ongoing trial but also supportive of the aims and methods of the trial.
Whether the DMEC will meet early in the trial:	If the DMEC members may meet early in the trial to discuss the protocol, the trial, the analysis plan, and structure, responsibilities and frequency of future activities and meetings. They may want to clarify any aspects with the investigators of the NONA-LISA study group.
Any issues specific to the disease under study:	Please see the NONA-LISA trial protocol for reference.
Any specific regulatory issues:	The DMEC should be aware of any regulatory implications of their recommendations.

Any other issues specific to the treatment under study:	Please see the NONA-LISA trial protocol for reference.
Whether members of the DMEC will have a contract:	A contract is not proposed. DMEC members should formally register their assent by confirming (1) that they agree to be on the Data Monitoring and Ethics Committee (DMEC) and (2) that they agree with the contents of this Charter.
**4. COMPOSITION**	
Membership and size of the DMEC:	The DMEC will consist of a small number of independent members with experience in clinical trials.<br>The independent committee members are not involved with the trial in any other way or have any competing interests that could impact the trial. Any competing interests, both actual and potential, should be declared. A short competing interest form should be completed and returned by the independent members to the trial coordinating centre (Annex 1).<br>The members of the DMEC for this trial are:<br>1. (1) Chair: Keith Barrington, Canada.<br>2. (2) Kajsa Bohlin, Sweden.<br>3. (3) Jakob Hjort, Denmark.
The Chair, how they are chosen and the Chair's role:	The Chair has expertise in clinical trials in neonatology. The NONA-LISA study group invited the Chair. The Chair is expected to plan the DMEC meetings and facilitate and summarise discussions.
Statistical assistance:	One of the members of the DMEC will serve as an external statistician. He/She will produce (or oversee the production of) the report to the DMEC and, if necessary, participate in DMEC meetings, guiding the DMEC through the progress report, participating in DMEC discussions and, on some occasions, taking notes.
The responsibilities of the NONA-LISA study group:	The NONA-LISA study group (e.g., the coordinating investigators) will provide input to the production of the non-confidential sections of the DMEC report.

The responsibilities of the coordinating investigator and other members of the NONA-LISA study group:	The coordinating investigators may be asked and should be available to attend open sessions of the DMEC meeting. The other NONA-LISA study group members will not usually be expected to attend but can attend open sessions when necessary (See Organisation of DMEC Meetings).
**5. RELATIONSHIPS**	
Relationships with the coordinating investigator, other trial committees (e.g., Trial Steering Committee (TSC), sponsor and regulatory bodies:	The trial protocols provide short statements of the committees' *responsibilities.*
Clarification of whether the DMEC are advisory (make recommendations) or executive (make decisions):	It is customary that the DMEC does not make decisions about the trial but rather makes recommendations to an appropriate executive committee (e.g., the NONA-LISA study group) or its Chair.
Payments to DMEC members:	No payments or rewards are specified.
The need for DMEC members to disclose information about any competing interests:	Competing interests should be disclosed. These are not restricted to financial matters – involvement in other trials or intellectual investment could be relevant. Although members may well be able to act objectively despite such connections, complete disclosure enhances credibility.<br>DMEC members should not use interim results to inform trading in pharmaceutical shares, and careful consideration should be given to trading in the stock of companies with competing products.
**6. ORGANISATION OF DMEC MEETINGS**	
Expected frequency of DMEC meetings:	The exact frequency of meetings will depend upon any statistical plans specified in the protocol and otherwise on trial events. Once

	recruitment begins, the DMEC plans to meet every 6-12 months to review safety and data quality.
Whether meetings will be face-to-face or by teleconference:	The first meeting should ideally be video conferencing to facilitate complete discussion and allow members to get to know each other. The DMEC will meet by video conferencing annually. Other meetings will be by email, teleconference, or video conferencing.
How DMEC meetings will be organised, especially regarding open and closed sessions, including who will be present in each session:	A mixture of open and closed sessions is recommended. Closed and open sessions should be defined. Only DMEC members and others they invite (e.g., a trial statistician) are commonly present in closed sessions.<br>The members of the study groups will be invited to attend the open sessions but at least the coordinating investigator (or delegate such as the trial sponsor) must attend the open sessions.<br>A person involved in the DMEC will take the minutes.
The format of the meetings should be as follows:	The online meetings may last for up to 60 minutes in total.<br>1. Open session: Introduction and any “open” parts of the report: The meeting will start with a presentation from the coordinating investigator (or delegate) of the progress in recruitment, as well as the demographic data (i.e., Table 1 of the manuscript) by study arm. This data can be circulated in an open report prior to the meeting.<br>2. Closed session: DMEC discussion of “closed” parts of the report (circulated in advance to the DMEC members only) that contains the list of outcomes (see below) and severe adverse events by allocated arm, tabulated by group with brief details about each SAE including relationship to intervention and outcome.<br>3. Open session: Discussion with other attendees on any matters arising from the previous session(s).<br>4. Closed session: extra closed session, if necessary
**7. TRIAL DOCUMENTATION AND PROCEDURES TO ENSURE CONFIDENTIALITY AND PROPER COMMUNICATION**	

Intended content of material to be available in open sessions:	Open sessions: Accumulating information related to recruitment and data quality (e.g., data return rates, treatment compliance) will be presented. Safety based on pooled data may be presented, at the discretion of the DMEC. Safety cases reported by the study groups will be reviewed on an individual basis.
Intended content of material to be available in closed sessions:	Closed sessions: In addition to all the material available in the open session, the DMEC will review safety data by allocation arm in the closed session of the meeting.
Who will see the accumulating safety data:	Only the members of the DMEC will see the accumulating safety data. The investigators will only be made aware of the results if new evidence emerges from other sources or if the trial provide “proof beyond reasonable doubt”.<br>DMEC members do **not** have the right to share confidential information with anyone outside the DMEC, including the coordinating investigator.
Who will be responsible for identifying and circulating external evidence (e.g., from other trials/systematic reviews):	Identification and circulation of external evidence (e.g., from other trials/systematic reviews) is not the responsibility of the DMEC members. The NONA-LISA study group will collate any such information.
To whom the DMEC will communicate the decisions/recommendations that are reached:	The DMEC will report its recommendations to the NONA-LISA study group. If the trial is to continue essentially unchanged, then it is often helpful for the report from the DMEC to include a summary paragraph.
Whether reports to the DMEC will be available before the meeting or only at/during the meeting:	The DMEC will receive the report one week before any meetings by email.
What will happen to the confidential papers after the meeting:	The DMEC members should store the papers safely after each meeting so they may check the following report against them. After the trial is reported, the DMEC members should destroy all reports.

8. DECISION MAKING	
What decisions/recommendations will be open to the DMEC:	There are no formal stopping rules. Possible recommendations may include the following:<br>● No action is needed; the trial continues as planned<br>● Early stopping due (e.g., to clear harm or benefit of the treatment or external evidence)<br>● Stopping recruitment within a subgroup<br>● Extending recruitment or extending follow-up<br>● Sanctioning or proposing protocol changes
The role of formal statistical methods, specifically which methods will be used and whether they will be used as guidelines or rules:	Analyses of safety and morbidity outcomes will be supplied to the DMEC along with any other analyses that the DMEC may request. The DMEC will consider safety every 6-12 months. The DMEC will also be given data quality reports at 6-12 monthly intervals.
How decisions or recommendations will be reached within the DMEC:	• The Chair will summarise discussions and encourage consensus; it may be best for the Chair to give their opinion last.<br>• The decision-making process includes whether there will be voting or other formal methods of achieving consensus. The method of deliberation should be kept from any overseeing committee as this may reveal information about the status of the trial's data.<br>It is recommended that every effort should be made for the DMEC to reach a unanimous decision. If the DMEC cannot achieve this, a vote may be taken, although details of the vote should not be routinely included in the progress report as these may inappropriately convey information about the state of the trial data. The trial's implications (e.g., ethical, statistical, practical, or financial) must be considered before any recommendation is made.
When the DMEC is quorate for decision-making:	Efforts should be made for all members to attend. The coordinating investigator will try to ensure a date is chosen to

	enable this. Members who cannot attend in person should be encouraged to attend by teleconference. If at short notice, one DMEC member cannot attend, the DMEC may still meet if at least the Chair (unless otherwise agreed), will be present. Suppose the DMEC is considering recommending significant action after such a meeting. In that case, the DMEC Chair should talk with the absent members as soon as possible to check whether they agree or disagree. If they disagree, a further teleconference should be arranged where all the DMEC members are present.
Can DMEC members who do not attend the meeting give input:	If the report is circulated before the meeting, DMEC members who cannot attend the meeting may pass comments to the DMEC Chair for consideration during the discussions.
What happens to members who do not attend meetings:	If a member does not attend a meeting, it should be ensured that the member is available for the second meeting. Otherwise, they should be asked if they wish to remain in the DMEC. If a member does not attend a third meeting, they should be replaced.
Whether different weights will be given to different endpoints (e.g., safety/efficacy):	The DMEC will focus on safety endpoints. (pain/discomfort, endotracheal intubation, mechanical ventilation, and death).
Any specific issues relating to the trial design that might influence the proceedings (e.g., cluster trials, equivalence trials, multi-arm trials):	No.
**9. REPORTING**	
To whom will the DMEC report their recommendations/decisions, and in what form:	This will by letter to the NONA-LISA study group, usually within three weeks. DMEC deliberations will be minute and stored at the trial office.

Whether minutes of the meeting will be made and, if so, by whom and where they will be kept:	Minutes from the closed and open sessions will be prepared by one of the DMEC members as agreed. Closed session minutes should be stored securely. The Chair should sign off any minutes or notes.
What will be done if there is a disagreement between the DMEC and the body to which it reports:	If the DMEC has serious problems or concerns with the NONA-LISA study group decision, a meeting between these groups will be held. The information to be shown will depend on the action proposed and the DMEC's concerns. Depending on the reason for the disagreement, confidential data will often have to be revealed to all those attending such a meeting. The meeting should be chaired by a senior member of the trial's office staff or an external expert who is not directly involved with the trial.
**10. AFTER THE TRIAL**	
Publication of results:	At the end of the trial, there may be a meeting to allow the DMEC to discuss the final data with principal trial investigators/sponsors and give advice about data interpretation. The DMEC may wish to see a statement that the trial results will be published in a correct and timely manner.
The information about the DMEC that will be included in published trial reports:	DMEC members should be named, and their affiliations listed in the main report unless they explicitly request otherwise. A summary of the timings and conclusions of DMEC meetings should be included in the body of this paper.
Whether the DMEC will have the opportunity to approve publications, especially for reporting any DMEC recommendation regarding the termination of a trial:	The DMEC may be allowed to read and comment on any publications before submission.
Any constraints on DMEC members divulging information about their	The DMEC may discuss issues from their involvement in the trial when permission is agreed with the NONA-LISA study group.

deliberations after the trial has been published:	
